# Identification of the parasite, *Trypanosoma cruzi*, in multiple tissues of epidemiological significance in the Virginia opossum (*Didelphis virginiana*): Implications for environmental and vertical transmission routes

**DOI:** 10.1371/journal.pntd.0010974

**Published:** 2022-12-19

**Authors:** Carson W. Torhorst, Zoe S. White, Chanakya R. Bhosale, Norman L. Beatty, Samantha M. Wisely

**Affiliations:** 1 Department of Wildlife Ecology and Conservation, University of Florida, Gainesville, Florida, United States of America; 2 Emerging Pathogens Institute, University of Florida, Gainesville, Florida, United States of America; 3 Division of Infectious Diseases and Global Medicine, Department of Medicine in the College of Medicine, Gainesville, Florida, United States of America; University of Texas at El Paso, UNITED STATES

## Abstract

**Background:**

*Trypanosoma cruzi*, a parasitic protozoan, is endemic to the Americas and the causative agent of Chagas disease in humans. In South America, opossums facilitate transmission via infected anal gland secretions in addition to transmission via triatomine vectors. In North America, the Virginia opossum is a reservoir host for the parasite with transmission routes that are not clearly defined. The unique biology of this marsupial provides the opportunity to investigate vertical transmission in this wildlife species *in situ*. Our objectives were to investigate alternative routes of transmission that may facilitate spillover into other species and to determine if vertical transmission was evident.

**Methodology/Principal findings:**

Virginia opossums were sampled at 10 trapping locations over a 10-month period in a 5-county region of north central Florida. Peripheral blood, fecal swabs, and anal gland secretions were collected from each adult individual, and peripheral blood was collected from joey opossums. Total DNA was extracted from each collected sample type, and *T*. *cruzi* infected individuals and the infecting Discrete Typing Unit (DTU) were identified using real time PCR methods. Adult Virginia opossums (n = 112) were infected with *T*. *cruzi* (51.8%, 95% CI [42.6–60.8%]) throughout the sampled period and at each location. *T*. *cruzi* DNA was found in each of the three biological sample types. Vertical transmission of *T*. *cruzi* was inferred in one litter of mother-dependent (n = 20, 5.0%, 95% CI [0.9–23.6%]) joey opossums where 2 joeys from this same litter were rtPCR positive for *T*. *cruzi*.

**Conclusions/Significance:**

We inferred vertical transmission from mother to neonate which may serve to amplify the prevalence of *T*. *cruzi* in adult Virginia opossums. *T*. *cruzi* DNA was detected in the anal gland secretions of Virginia opossums. Infected anal gland secretions suggest a possible environmental route of transmission for *T*. *cruzi* via the deposition of contaminated feces and spraint at wildlife latrines. Only DTU1 was identified in the sampled population which is consistent with human autochthonous cases in the United States.

## Author summary

The etiological agent of Chagas disease is the hemoflagellated protozoan, *Trypanosoma cruzi*. The classical route of infection is via the bite of triatomine bugs. In the southern two thirds of the United States, the Virginia opossum (*Didelphis virginiana*) serves as a key reservoir host in the vector-borne transmission cycle of *T*. *cruzi*. Independent of the vector, Virginia opossums may maintain and transmit the parasite through vertical and alternative horizontal transmission pathways. In this study we observed vertical transmission in opossums which may amplify the natural infection prevalence. In the South American opossum species, *Didelphis marsupialis*, the anal gland secretions, when infected with *T*. *cruzi*, are known to cause infection in other mammalian hosts. In Virginia opossums, it is possible that this evolutionary relationship also exists; we identified *T*. *cruzi* present in anal gland secretions in the sampled Virginia opossums of Florida which may have important disease transmission implications. These findings provide evidence that the anal gland secretions of the Virginia opossum are infected with *T*. *cruzi*; however work remains to be done to determine if anal gland secretions are infectious and provide an alternative route of transmission to wildlife and potentially humans.

## Introduction

*Trypanosoma cruzi*, a kinetoplastid protozoan, is the causative agent of Chagas disease (CD) in humans, and vectored by triatomine bugs [1,2]. Across the United States (US), CD is an emerging and likely underdiagnosed infectious disease; only 78 cases of autochthonous Chagas have been diagnosed in the US since 1950 [3–6]. Despite the low prevalence in people, the parasite has been shown to infect 27 different mammal species in the US which serve as reservoir hosts [1,7–9]. Chagas is traditionally understood as a vector-borne disease, however, environmental and vertical transmission have been observed [1,5,6,8,10,11]. Vertical transmission is a known to occur in both humans and domestic dogs, however, its relative impact on the sylvatic transmission cycle is unknown [2,12]. Environmental transmission via the ingestion of food products contaminated with infected triatomine fecal matter is an increasingly recognized route of transmission [13]. This transmission route has caused large zoonotic *T*. *cruzi* outbreaks in Brazil, Venezuela, and other South American countries [13–17]. Consumption of an infected kissing bug is an additional horizontal route that impacts domestic reservoirs, like dogs, and this route is presumed to be one way wildlife are infected with the parasite [7,18–22].

Opossum species are also suspected to maintain independent, alternate transmission cycles of *T*. *cruzi* that do not involve the arthropod vector [23,24]. It has been shown that the parasite will reproduce and transition to its metacyclic trypomastigote, thus completing the life cycle of the parasite within the anal gland secretions of certain opossum species [23,24]. This life cycle has been confirmed in *Didelphis marsupialis*, a South and Central American opossum species [22,23,25–28]. *T*. *cruzi* infected anal gland secretions of *D*. *marsupial* have been shown to be infectious in a laboratory setting, providing strong evidence for an independent sylvatic transmission cycle [23,24,26,29]. In North America, the parasite has been found in anal glands of *D*. *virginiana*, but the prevalence of infected secretions and its ability to infect other mammals is unknown. It is, however, suspected that *D*. *virginiana* may potentially transmit *T*. *cruzi* to other reservoir hosts [5,22].

In addition to their suspected role in pathogen transmission, the Virginia opossum, a marsupial, provides the unique opportunity to observe vertical transmission of *T*. *cruzi in situ*. Developing neonates and juveniles (joeys) can be observed within their mother’s marsupium for up to 70 days [30–32]. Within the marsupium, joey development can be split into two time periods; the mother-dependent stage (50 to 65 days post-parturition), where the joeys are completely dependent on the mother and cannot freely exit the marsupium, and the weaned stage, where the joeys can freely exit the marsupium and enter the external environment independent from the mother [30–32]. While the joeys are dependent on their mother, the marsupium largely isolates them from the external environment which provides the opportunity to identify vertical transmission as the most parsimonious explanation for their infection. Even though *T*. *cruzi* is known to be transmitted vertically in mammals, little is known about how this mode of transmission impacts wildlife populations [8,33–35]. By identifying vertical transmission, we also gain insight into how the multiple modalities of *T*. *cruzi* transmission may maintain or amplify prevalence of the parasite within this reservoir host species.

Despite the known status of the Virginia opossum as a *T*. *cruzi* reservoir host, there are still questions that surround the epidemiological and ecological role opossums play in the transmission cycle of *T*. *cruzi*. The objectives of this study investigated the role of Virginia opossums in the transmission cycle of *T*. *cruzi* in Florida by determining: (a) if the anal gland secretions of opossum are infected with *T*. *cruzi* and (b) if vertical transmission is taking place between infected mother and her joeys. We also confirmed the association between opossums and the *T*. *cruzi* Discrete Typing Unit, DTU1 (TcI). In addition, we determined the pathogen prevalence across 3 epidemiologically relevant sample types collected from Virginia opossums.

## Methods

### Ethics statement

Scientific collecting permits were obtained from the Florida Fish and Wildlife Conservation Commission and permission was obtained from the appropriate land managing agencies or private landowners to gain access to the locations where live trapping took place. All animal trapping, handling, and sample collection methods were approved by the University of Florida Institutional Animal Care and Use Committee (IACUC Study #202011195) and the Florida Fish and Wildlife Conservation Commission.

### Locations of sample collections

**Fig 1:**
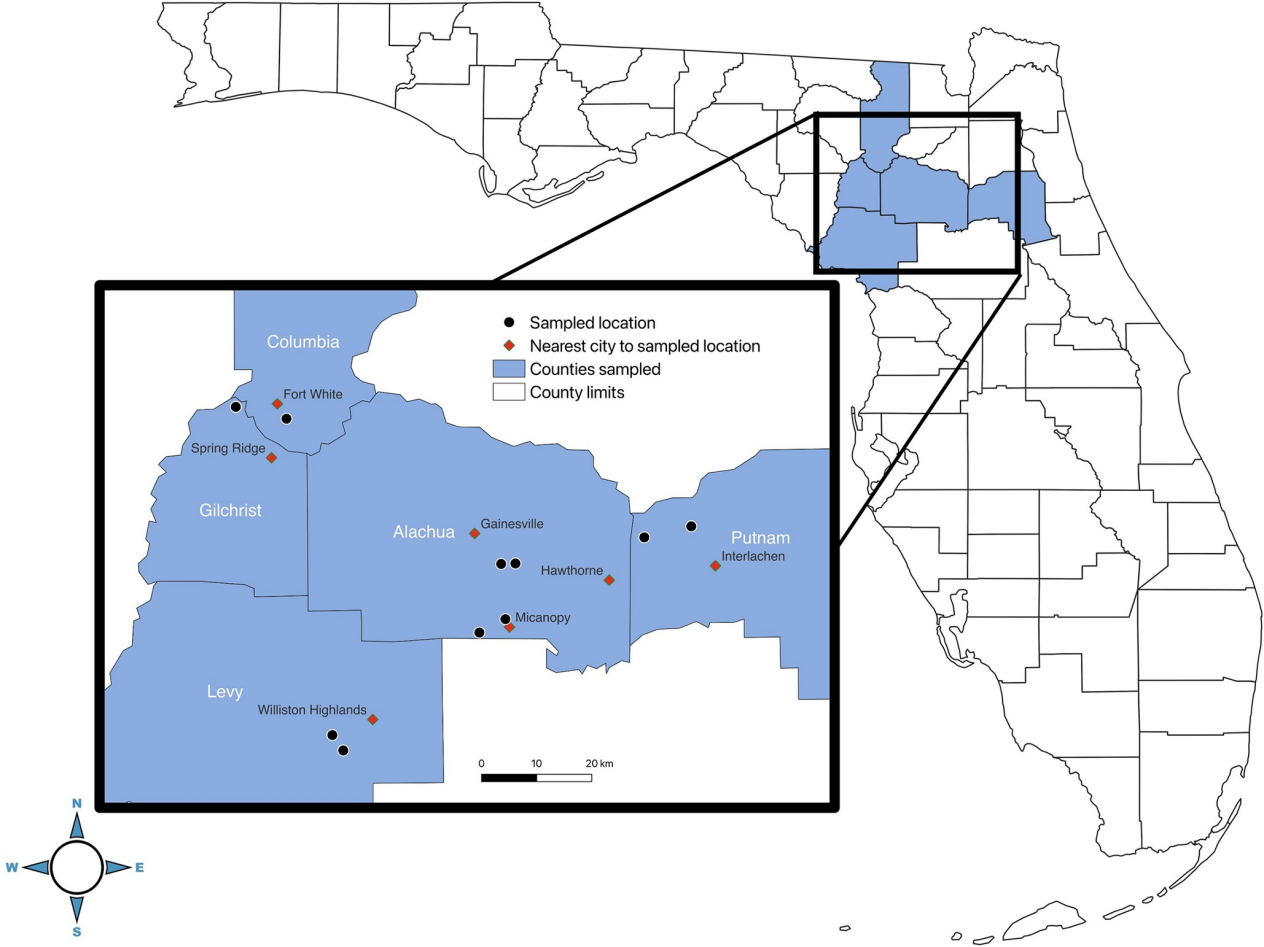
Identification of the locations where Virginia opossums were sampled. Identification of the 5 counties, sampled location, and nearest city to the sampled location in north central Florida where opossums were live trapped. Map was created using QGIS Geographic Information System version 3.22.5-Białowieża, http://qgis.osgeo.org. County layers map (TIGER/Line Shapefile, 2016, state, Florida, Current County Subdivision State-based) and city locations (TIGER/Line Shapefile, Current, State, Florida, Places) were accessed from the United States Census Bureau, https://catalog.data.gov/dataset.

Virginia opossums were live trapped at 10 locations across 5 north central Florida counties between January 2021 to October 2021 (Fig 1). Each trapping location was selected based on the identification of triatomine intrusion into or domiciliation of households, triatomines observed in close proximity to a home (e.g., stacked building lumber), or locations in close (<2km) proximity to a housing location where triatomines were identified. Locations consisted of multiple land uses including private property and state managed lands across multiple habitat types in the Gulf Coast Forest ecosystem: hardwood hammock, hardwood swamp, mixed hardwood-pine forests, natural pine forests, and human modified peridomestic habitats.

### Animal handling and processing

At each sampling site we conducted 8 to 12 nights of trapping using 20 or 21 Tomahawk live traps (series 106 and 207) (Tomahawk Live Trap, Tomahawk, WI). Traps were opened each evening approximately one hour before sunset and baited using both wet and dry cat food. Each set trap was checked the following morning. All traps were closed and unavailable for trapping during daylight hours. Opossums were chemically immobilized using a combination of 10mg/kg Ketamine and 2mg/kg Xylazine [36]. Each animal was temporarily marked using hair dye, and any recaptured individuals were immediately released at the location of capture. Any individual that was chemical immobilized was allowed to fully recover prior to release at the original location of capture. Body mass, body condition, and all external injuries were recorded and each animal was inspected for ectoparasites and anal gland self-expression. Each adult female opossum’s marsupium was completely searched to identify the number of joeys held by each female.

### Field sample collection

After immobilization, peripheral blood, fecal swab, and anal gland secretion samples were collected. Blood samples were collected by puncturing the skin between the middle digits of the forepaw with a medical lancet and blood was captured on a Nobuto strip (Sterlitech Cooperation, Auburn, WA, USA). Blood samples were also collected from joey opossums found within the marsupium or attached to the mother opossum, and their age class was determined as mother-dependent or weaned. Mother-dependent joeys were held within the marsupium, their eyes were still underdeveloped and firmly closed, and they were still firmly attached to the mothers teat with no sign of independence from the mother [30,32]. Weaned joeys had fully developed eyes, ears, and hair, they were able to freely detach from the teat, and could exit the marsupium under their own power [30,32]. The number of joeys within the mother’s marsupium were recorded, and blood samples were collected from one half of the tallied individuals to reduce any impact that handling may have had on the litter. Joey blood was collected by puncturing the paw pad using a fetal lancet and captured on a Nobuto strip. Nobuto strips were air dried and stored at room temperature until DNA could be extracted.

Only peripheral blood was collected from joeys; from subadults and adults, fecal and anal gland samples were taken in addition to peripheral blood. Fecal swabs were collected from adult opossums by gently inserting a fecal loop and FLOQ swab into and past the anus to the colon (COPAN Diagnostics, Murrieta, CA, USA). The FLOQ was then used to gently swab the colon of each opossum for 10 seconds, and then placed in 600 uL of Gentra PureGene cell lysis solution (Qiagen Puregene, Qiagen, Hilden, Germany). Anal gland secretions of each adult opossum were expressed by applying light pressure to the perianal region of anus. The expressed secretion was collected using a FLOQ swab and placed into 600 uL of Gentra PureGene cell lysis solution. These secretions were always expressed after the fecal sample was collected so these secretions did not contaminate the fecal swab taken. Care was taken to exclude fecal contamination from the anal gland secretion by only collecting the expressed green secretion that directly came from the anal gland of the sampled opossum. Both the fecal swab and anal gland secretions were stored at room temperature until the DNA from each sample was extracted. Finally, it was recorded when any individual became agitated and expressed their anal glands in a defensive behavior as the trap was approached. Anal gland expression was identified visually by presence of the green anal gland secretion in the perianal region of sampled opossums.

### DNA extraction

DNA was extracted from the peripheral blood, fecal swab, and anal gland secretion samples collected using a Gentra PureGene extraction kit following the manufacturer’s protocol with slight modifications for each sample type (Qiagen Puregene, Qiagen, Hilden, Germany). Peripheral blood, collected using a Nobuto strip, was subject to 30-minute incubation period in 300 uL of red blood cell lysis solution to remove the red blood cells still bound to the Nobuto strip. Each Nobuto strip was then incubated for 24 hours in 600 uL of cell lysis solution at 56°C to lyse the buffy coat cells bound to the Nobuto strip. Finally, 10 uL of Proteinase K (ProtK) was added to each sample containing cell lysis solution and Nobuto strip. The sample was then incubated at 56°C for 24 hours to digest the proteins within the extraction solution (Themo Fisher Scientific, Waltham, MA). Following ProtK digestion, total DNA from the peripheral blood sample was extracted following the manufacturer’s recommended protocol. Pelleted DNA was resuspended in 50 uL in AE buffer and stored at -20°C until amplification. For the fecal swab and anal gland secretions samples, 200 uL of the original 600 uL field collected sample solution was placed in 400 uL of cell lysis solution. These samples were incubated in the cell lysis solution containing the sample at 56°C for 48 hours, and further digested at 56°C for 48 hours with 10 uL of ProtK added each 24-hour period during the stage. Total DNA was extracted similarly to the blood sample. Pelleted DNA was suspended in 100 uL of AE buffer. All extracted DNA was quantified using a NanoDrop 2000 (Themo Fisher Scientific, Waltham, MA) and diluted to 20ng/uL and stored at -20°C.

### *Trypanosoma cruzi* detection and DTU determination

To identify *T*. *cruzi* infected individuals, we used a quantitative-PCR (q-PCR) assay which detects the nucleic acid of the parasite. A 166bp segment of *T*. *cruzi* satellite DNA (satDNA) was amplified with a q-PCR using a 2x Roche Fast Start universal master mix with the primers Cruzi1, Cruzi2, and Cruzi3 PrimeTime 5’ 6-FAMTM/ZENTM/3’ IBFQ (IDT, Coralville, Iowa) following previously described methods [37,38]. The 166bp satDNA segment was from the 195bp tandem repeating satDNA unit that has been used as a molecular diagnostic tool to identify *T*. *cruzi* in infected wildlife and triatomines [22,39–41]. In each reaction, 2 uL of host template (20 ng/uL) was added to the master mix with a separate exogenous amplification control which used the fluorophore VIC (10x Exo IPC Mix, 50 IPC Exo, Thermo Fisher Scientific, Waltham, MA). The exogenous amplification control was used in individual assays to identify host template DNA dilutions that contained amplification inhibitors and could create false negatives. Each plate of assays was performed with a negative control (blanked with molecular grade water) to determine if contamination occurred in the laboratory, an exogenous amplification positive control (containing molecular grade water and exogenous amplification control), and a positive control dilutions series from a cell culture isolate of TcI. Precautions were taken to exclude contamination by the positive control by storing positive controls and master mix reagents separate from opossum gDNA samples. Amplification did not occur in any negative controls. All amplifications were performed on an ABI 7500 FAST Real Time PCR machine (Thermo Fisher Scientific, Waltham, MA).

A sample was classified as *T*. *cruzi* positive when the Ct value was ≤ 38 [42]. Any sample that amplified between the Ct values of 38 to 40 was labeled as suspect positive, and these samples were rescreened [42]. For those samples where the exogenous amplification control did not amplify, they were rescreened at a template DNA dilution series of 1:5 and 1:10 to reduce amplification inhibition within the sample. *T*. *cruzi* positive samples were typed for DTU’s (TcI-TcIV) via a multi-stepped q-PCR approach following previously described methods [43]. We modified the DTU identification protocol as follows: FAM was used to identify TcI (0.15uM) (Affinity Plus Affinity Plus 5’ 6-FAMTM / 3’ BHQ-1), Cy5 to identify TcIII (0.1uM) (Affinity Plus 5’ Cy5 / 3’ IBRQ), Cy3 was used as the florescent probe for TcIV (0.3uM) (PrimeTime 5’ Cy3TM / 3’ IBRQ), and Yak-yellow was used to identify TcIII/TcV/TcVI (0.1uM) (Affinity Plus 5’ YAK / 3’ IBFQ). DTU identification was discontinued after 3 unsuccessful amplification attempts.

We inferred that the presence of nucleic acid of *T*. *cruzi* was indicative of an infected individual [22,39–41]; however we were unable to infer whether or not that biological material was infectious. We inferred vertical transmission when an infected joey opossum was both dependent on their mother and their mother was infected with the parasite. To be considered a mother-dependent litter each individual in the litter had to meet the following criterion: the joeys’ eyes were still underdeveloped and firmly closed, their ears, hair and whiskers had not yet fully developed, and they were still firmly attached to the mothers teat with no sign of independence from the mother. A violation of any one of these criteria excluded them as a mother-dependent litter. An individual was considered *T*. *cruzi* infected when any one of the three independently collected sample types (peripheral blood, fecal swab, or anal gland secretions) contained *T*. *cruzi* nucleic acid. To calculate apparent prevalence, the number of *T*. *cruzi* infected individuals was divided by the total number of individuals sampled. A Wilson 95% Confidence Interval (CI) was calculated using EPI tools as a categorical variable and was reported for each prevalence [44,45].

## Results

In total, samples were collected from 198 Virginia opossums (n = 112 adults; n = 86 joeys). Triplet samples of peripheral blood, fecal swabs, and anal gland secretion were collected from each adult, and from the adults sampled, 58 had at least one independent sample type infected with *T*. *cruzi* (51.8%, 95% CI [42.6%– 60.8%]) (Tables 1 and 2). Infected adults were found in each county where opossums were sampled (Table 1). The apparent prevalence varied between counties where the highest prevalence was in Columbia County (n = 13) (84.6%, 95% CI [57.8–95.7%]) and the lowest was in Putnam County (n = 23) (39.1%, 95% CI [22.2–59.2%]) (Table 1). Each county varied in the number of adults sampled, the smallest adult sample size was in Columbia County (n = 13) and the largest was from Alachua County (n = 41) (Table 1).

**Table 1:** Infection prevalence of *Trypanosoma cruzi* by county for adult Virginia opossums. A total of 112 Virginia opossums were sampled.

Location:	Adults Infected / Sampled	Prevalence (Adults Infected / Sampled) [Wilson 95% CI]
Alachua County	21 / 41	51.2% [36.5–65.7%]
Columbia County	11 / 13	84.6% [57.8–95.7%]
Gilchrist County	9 / 17	52.9% [31.0–73.8%]
Levy County	8 / 18	44.4% [24.6–66.3%]
Putnam County	9 / 23	39.1% [22.2–59.2%]
Total:	58 / 112	51.8% [42.6–60.8%]

**Table 2:** Number of individuals infected and prevalence of *Trypanosoma cruzi* by sample type. Average C_T_ value, maximum and minimum CT value for rtPCR assays are given in parentheses for each tissue type.

Prevalence by Sample type:	Peripheral blood (C_T_, max, min)	Fecal swab C_T_, max, min)	Anal Gland Secretion C_T_, max, min)	Total No. infected individuals:	Prevalence (Total No. infected individuals / 112) [Wilson 95% CI]
No. of infected adult individuals for each sample type:	57 (23.943, 37.385, 16.307)	7 (33.744, 37.898, 21.839)	21 (34.105, 38.371, 25.862)	58	51.8% [42.6–60.8%]
No. of individuals with six combinations of infected tissue types	+	–	–	33	29.5% [21.8–38.5%]
	–	+	–	0	0% [0–3.3%]
	–	–	+	1	0.9% [0.2–4.9%]
	+	+	–	4	3.6% [1.4–8.8%]
	–	+	+	0	0.0% [0–3.3%]
	+	–	+	17	15.2% [9.7–23.0%]
	+	+	+	3	2.7% [0.9–7.6%]

In total, 86 joey opossums were sampled from 25 litters in this study: from 20 litters, 73 joeys were sampled that were dependent on their mother (mother-dependent), and from 5 litters, 13 joeys were sampled that had been weaned from the mother. The largest number of joeys sampled was in Alachua County (n = 41) and no joeys were sampled in Gilchrist or Putnam County. Two of 73 mother-dependent joeys (2.7%, 95% CI [0.8% - 9.5%]) (CT values = 33.644, 35.327) from 1 of 20 litters (5.0%, 95% CI [0.9–23.6%]) were infected with *T*. *cruzi*, both of whom were from the same *T*. *cruzi* infected mother. Also, 2 weaned joeys (15.4%, 95% CI [4.3%– 42.2%]) (CT values = 30.853, 34.834) from 1 litter (20.0%, 95% CI [3.6–62.4%]) were infected with *T*. *cruzi* and both were from the same mother who was also infected with *T*. *cruzi*. Each pair of infected sibling joeys came from different infected mother opossums. From the sampled marsupial pouches, there was no observations of triatomine intrusion into or triatomine fecal matter within the marsupium.

**Fig 2:**
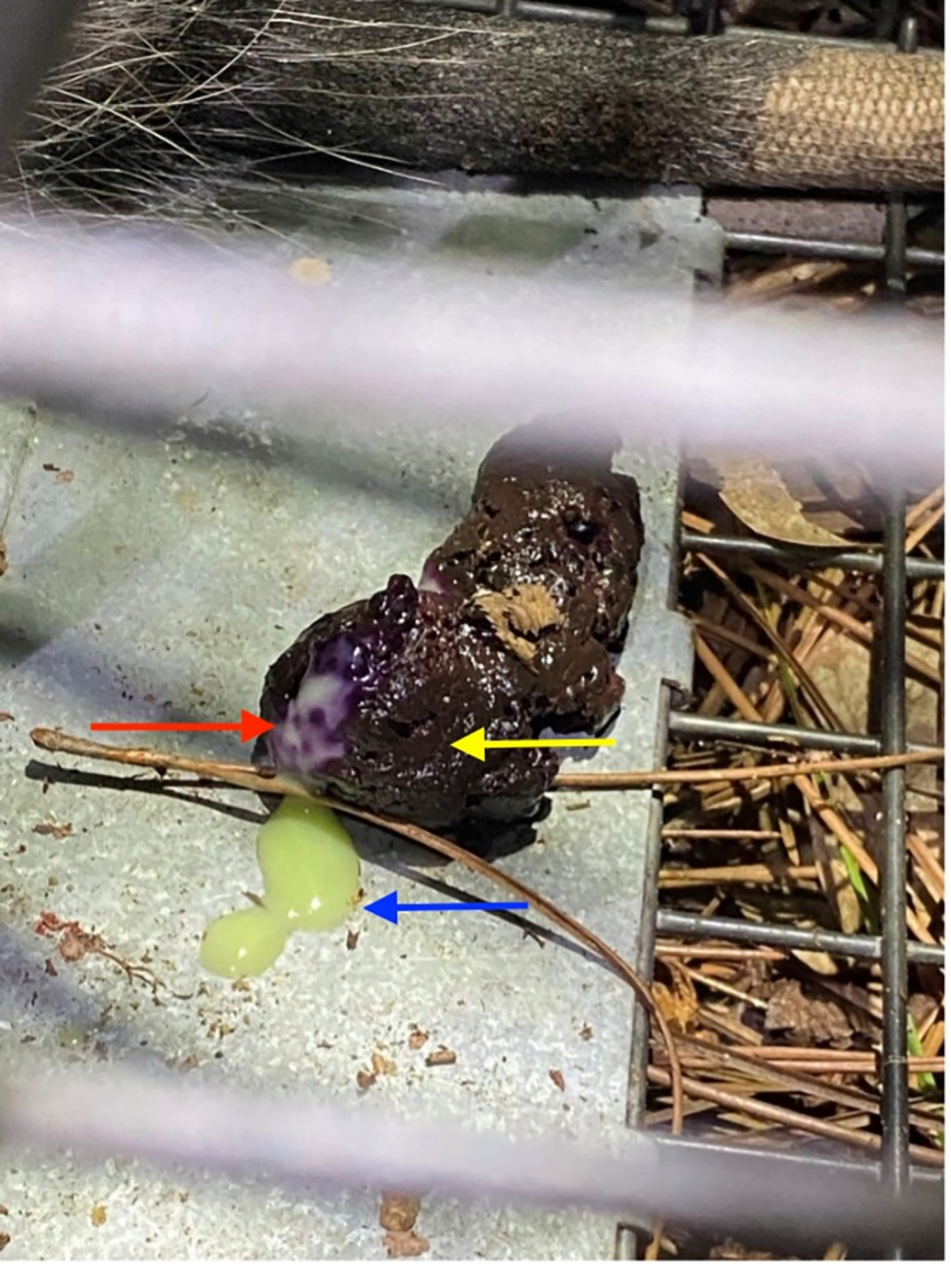
Opossum feces and anal gland secretion. Image depicting opossum feces (yellow arrow) contaminated with anal gland secretion (red arrow). Opossum anal gland secretion (blue arrow) can be seen in close association with the fecal bolus that is sitting on the trigger pan of the Tomahawk livetrap. This individual was sampled but negative for *T*. *cruzi*. The tail of the Virginia opossum can be seen in the background of the image. (Photo credit: C. W. Torhorst).

Across the triplet of sample types collected, there were individuals that had only one *T*. *cruzi* infected sample type and individuals that had a combination of *T*. *cruzi* infected sample types (Table 2). Of the 58 infected opossums, 57 had *T*. *cruzi* DNA detected in peripheral blood; the remaining individual was positive for *T*. *cruzi* in the anal gland secretion but in no other sample type. In the field, we observed anal gland secretions contaminating opossum feces in the trap, due to anal gland expression during defecation (Fig 2). Also, as the trap was approached, 30 of the 112 adults expressed their anal gland secretions (26.8%), and of this 30, 3 of those individuals had *T*. *cruzi* infected anal gland secretions (10%); however, none of those individuals had *T*. *cruzi* positive fecal swabs. TcI was the only DTU identified in any of the samples; of the 57 positive blood samples, 55 were typed as TcI (96.5%), of the 21 positive anal gland secretions 7 were typed as TcI (33.3%), and of the 7 individuals with positive fecal swabs 6 were typed as TcI (85.7%). One joey opossum was positive for TcI (1/4, 25%). All other samples were unable to be DTU typed.

## Discussion

In Florida, 51.8% of Virginia opossums were infected with *T*. *cruzi* across 5 Florida counties (Table 1) (Fig 1). The prevalence observed in this study was consistent with a previous study from Florida that found a 52% prevalence of exposure to *T*. *cruzi* using serological methods [7,9]. In this study, we sampled a total of 198 Virginia opossums, which included 112 adult and 86 joeys (Table 1). The only DTU identified in the infected population was TcI which supports the previously hypothesized association between Virginia opossums and this genotype [22,46–50]. In total 57 of the 112 adult opossums sampled had *T*. *cruzi* infected peripheral blood samples (50.9%), 7 had infected fecal swabs (6.3%), and 21 had infected anal gland secretions (18.8%) (Table 2). Each infected sample type has an epidemiological consequence. Infected peripheral blood likely indicates that the individual was actively circulating the parasite thereby creating the opportunity for a vector to ingest a *T*. *cruzi* trypomastigote upon taking a blood meal from the infected host and continuing the transmission cycle in the triatomine vector [28]. Fecal swabs were also found to be infected with *T*. *cruzi* which indicates expulsion of the parasite from the digestive tract. *T*. *cruzi* is known to infect the epithelial layers of the large intestine and colon and is shed along with the epithelial cells in feces [22,50]. Alternatively, *T*. *cruzi* from feces may have come from ingested and infected triatomine bugs. Virginia opossum anal gland secretions were also infected with *T*. *cruzi*, and opossums were observed self-expressing their anal glands which would also introduce these potentially infectious secretions to the surrounding environment.

In total, 2 of the 73 sampled mother-dependent joeys (2.7%) were infected with *T*. *cruzi* which occurred in 1 of the 20 sampled litters of mother-dependent joeys. The likely transmission of *T*. *cruzi* vertically to joey opossums adds to the growing list of pathways through which *T*. *cruzi* is maintained on the landscape in wildlife reservoirs. Because the apparent prevalence among neonates was low, we infer that vertical transmission is not solely maintaining the high prevalence seen in adults (51.8%); rather, vertical transmission is likely amplifying the prevalence of vector-infected individuals thus increasing the number of infected adult opossums on the landscape independent from vector-borne transmission. The observed prevalence of vertical transmission in mother-dependent joeys is consistent with prevalence observed in human cases of congenital transmitted *T*. *cruzi* (~5%) [34].

**Fig 3:**
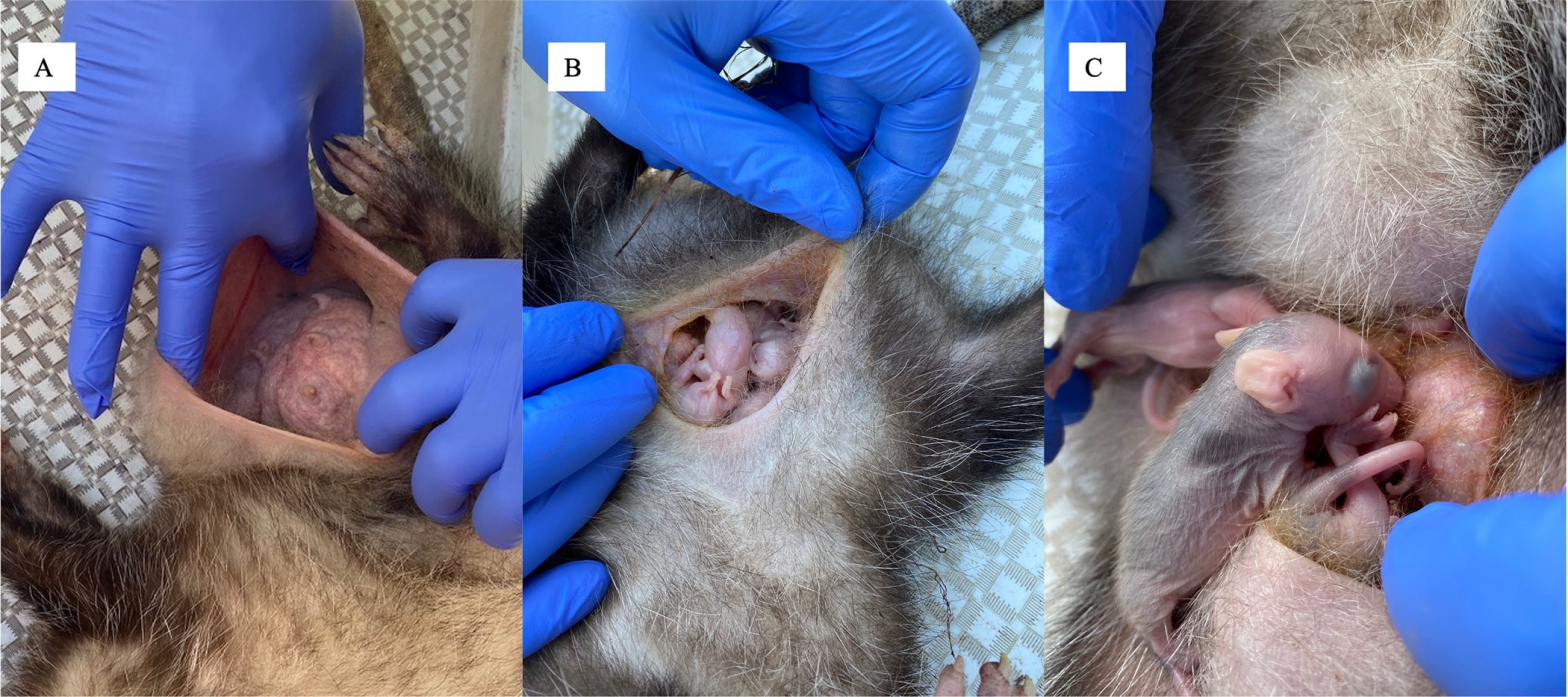
Female Virginia opossum and joeys. A depiction of the marsupium from two adult female Virginia opossums. From the first female, her marsupium, which is expanded to show that it does not contain joeys, and to further detail the cleanliness of the feature and the expanse of the pouch that holds the joeys within (A). From a second female her marsupium gently expanded to expose the mother-dependent joeys held within (B), and from a third female, a mother-dependent joeys with its categorically important features: developing hair, ears, eye openings, while still attached to the mother’s teat (C) (Photo credit: C.W. Torhorst).

The role of the marsupium is to isolate neonate joeys from the external environment and therefore likely excludes triatomines from the marsupium as joeys progress through their stages of fetal development (Fig 3) [30]. We did not observe triatomines or their fecal matter in the marsupium of the sampled adult females, however, we cannot exclude the possibility that a triatomine entered the marsupium and infected a neonate, as triatomines were observed in the sampled locations. Neonates were too young, however, to ingest a triatomine bug (Fig 3). These observations and inferences support vertical transmission as the most parsimonious explanation for the infection status of the mother-dependent joeys (Fig 3). Whether vertical transmission is taking place in other placental reservoirs like raccoons and armadillos, as it does in humans, is still unknown but could occur.

The anal gland secretions of opossums were found to be infected with *T*. *cruzi* (18.8%) (Table 2). This infection is consequential because there is strong evidence in other opossum species that this secretion contains the infective life stage of *T*. *cruzi*, the metacyclic trypomastigote [22–24,27]. These secretions were also observed contaminating the perianal region and deposited fecal matter of opossums in our study (Fig 2). Fecal swabs also tested positive for the presence of *T*. *cruzi* (6.3%) (Table 2) but it is not clear if *T*. *cruzi* nucleic acid in feces came from the sloughing of infected host epithelial tissue in the feces or from the consumption of infected vectors. Regardless, it is important to consider that fecal deposits and anal gland spraints are a potential source for environmental transmission. Indeed, it is clear that *T*. *cruzi* can persist in the environment as shown from investigations into fruits and fruit juices contaminated with infected triatomine feces leading to mass transmission outbreaks in South and Central America [13,51]. Further investigation is needed to confirm opossum excretions as hazards for transmission.

In this study we provide evidence that the Virginia opossum is an amplifying reservoir for *T*. *cruzi* due to its complex relationship with the parasite. This relationship impacts the way in which the parasite is maintained on the landscape and provides potentially novel and underappreciated routes of transmission. In Florida, we found that the Virginia opossum is infected with *T*. *cruzi* across a variety of habitats and is likely capable of shedding the parasite into the environment. Given the synanthropic habits of opossums and their close proximity to human dwellings, their fecal latrines and anal spraints could serve as a transmission hazard to people. Vertical transmission of *T*. *cruzi* likely amplifies the prevalence of the parasite separate from the vector in Virginia opossums. Vertical transmission is not well understood in wildlife but should be considered as an amplifying factor for transmission among reservoir species. In conclusion, vertical transmission in coordination with the infective anal gland secretions of Virginia opossum increases the complexity and adds to our knowledge around how opossums maintain *T*. *cruzi* infection in Florida.
